# Stratification overcomes ABA-mediated seed dormancy by uncoupling RGL2/ABI5 inhibition from α-amylase expression

**DOI:** 10.1007/s44154-026-00312-6

**Published:** 2026-06-22

**Authors:** Yuan Tian, Qin-Lai Liu, Mo-Xian Chen, Ying-Gao Liu

**Affiliations:** 1 State Key Laboratory for Development and Utilization of Forest Food Resources, State Key Laboratory of Tree Genetics and Breeding, The Southern Modern Forestry Collaborative Innovation Center, College of Life Sciences, Nanjing Forestry University, Nanjing, China; 2 College of Life Science, Shandong Agricultural University, Tai‘an, China; 3 State Key Laboratory of Green Pesticide, Center for Research and Development of Fine Chemicals, Guizhou University, Guiyang, Guizhou 550025 China; 4 School of clinical and Basic Medical Sciences, Shandong First Medical University & Shandong Academy of Medical Sciences, Jinan, Shandong 250000 China

**Keywords:** Stratification, Abscisic Acid (ABA), Gibberellins (GA), Seed dormancy, *RGL2*, *SLEEPY1*, *CYP707A2*, α-amylase

## Abstract

Cold-pretreatment (stratification) is widely employed to overcome seed dormancy and enhance germination, however, its underlying molecular mechanisms remain elusive. We tested the hypothesis that stratification alleviates the repression of α-amylase expression, a critical step for endosperm starch mobilization during germination. Stratification at 4 °C effectively overcame dormancy in wild-type *Arabidopsis thaliana*, the ABA-catabolism mutant *cyp707a2*, the GA-insensitive mutant *sleepy1*, and the *cyp707a2 sleepy1* double mutant, but failed to rescue the dormancy phenotype of the GA-biosynthesis mutant *ga3ox1*. Moreover, stratification markedly promoted starch hydrolysis by increasing both the transcript abundance and the enzymatic activity of α-amylase in freshly harvested seeds of wild-type and in those mutants exhibiting elevated endogenous ABA or treated with exogenous ABA. Concomitantly, stratification substantially suppressed the expression of *RGL2*, a DELLA protein that represses GA signaling, and *ABI5*, a key ABA-responsive transcription factor. Collectively, our data indicates that stratification initiates GA biosynthesis, thereby relieving RGL2-mediated repression of α-amylase expression. Secondly, stratification mitigates ABA-imposed inhibition of germination without alleviating the suppressive effect of ABA on post-germinative seedling development.

**Supplementary Information:**

The online version contains supplementary material available at 10.1007/s44154-026-00312-6.

## Introduction

Seed germination is a multifaceted developmental program that initiates with water imbibition by the quiescent dry seed and culminates in the elongation of the embryonic axis. This process is orchestrated by a network of endogenous and exogenous factors, including the phytohormones abscisic acid (ABA), gibberellins (GAs), and ethylene, the gaseous signaling molecules nitric oxide (NO) and reactive oxygen species (ROS), and environmental variables such as light intensity and temperature (Jurdak et al. 2022; Yang et al. 2020a, b; Zhao et al. 2024). Temperature constitutes a pivotal environmental determinant governing seed dormancy and germination. Cold-pretreatment, which is also called stratification, is widely employed to overcome seed dormancy (Ravindran et al. 2017; Zhang et al. 2023a, b, c). This treatment enhances both the percentage and rate of germination in *Echinacea* spp., *Arabidopsis thaliana*, *Pyrus malus* (apple), and *Corylus avellana* (hazel) (Andriotis et al. 2005; Liang et al. 2020; Macchia et al. 2001; Saisho et al. 2011; Yamauchi et al. 2004). Phytohormones such as ABA, GAs, and ethylene are implicated in temperature-mediated dormancy release and subsequent germination. Additionally, genes such as DOG1 and CBF have been reported to modulate this process (Kendall et al. 2011). Nevertheless, the precise molecular mechanisms underlying temperature-regulated dormancy release and germination remain elusive.

GAs are essential phytohormones that orchestrate a broad spectrum of developmental processes, including seed germination, stem elongation, leaf expansion, floral transition, and seed maturation (Ali et al. 2022; Hu et al. 2021; Prasetyaningrum et al. 2021; Zhang et al. 2023a, b, c). In *Arabidopsis thaliana*, GAs are proposed to exert two principal functions during germination. First, they promote radicle emergence by facilitating the weakening of the tissues surrounding the embryo. Second, they enhance the embryo's growth potential, evidenced by the diminished elongation rate of GA-deficient embryos (Hauvermale and Steber 2020; Ogawa et al. 2003). GA-deficient mutants, such as *ga1* and *ga2*, exhibit strong seed dormancy and fail to germinate in the absence of exogenous GA treatment (Otsuka et al. 2004; Shu et al. 2016). Pharmacological inhibition of GA biosynthesis with paclobutrazol (PAC) or uniconazole markedly suppresses seed germination in *Arabidopsis thaliana* (Lee et al. 2022; Mangano et al. 2023). GAs play a critical role in regulating temperature-dependent seed dormancy and germination processes (Maupilé et al. 2024). Stratification has been shown to modulate tissue sensitivity to GAs in *Arabidopsis thaliana* seeds (Chen et al. 2024; Jurdak et al. 2022). Elevated GA levels occur concomitant with dormancy release during seed imbibition and stratification. In dormant seeds, treatment with exogenous GA exploits this mechanism to release dormancy, thereby accelerating seedling establishment (Jurdak et al. 2022). DELLA proteins constitute a subfamily within the GRAS (GAI, RGA, and Scarecrow) transcription factor family, acting as nuclear-localized negative regulators of GA signaling that operate immediately downstream of GA receptors to regulate diverse facets of GA-mediated growth and development (Dahal et al. 2025; Pysh et al. 1999). GA alleviates DELLA-mediated repression during germination, stem elongation, and floral transition by promoting DELLA protein degradation through the ubiquitin–proteasome pathway (Huang et al. 2024; Islam et al. 2025; Xu et al. 2021). In *Arabidopsis thaliana*, five DELLA proteins with partially redundant functions mediate GA signaling responses including REPRESSOR OF GA (RGA)、GIBBERELLIC ACID INSENSITIVE (GAI)、RGA-LIKE1 (RGL1)、RGA-LIKE2 (RGL2) and RGA-LIKE3 (RGL3). RGL2 plays a predominant role in germination regulation while RGA, GAI, and RGL1 contribute to a lesser extent (Islam et al. 2025; King et al. 2001). GID1, a GA receptor, enhances the interaction between RGL2 and the F-box protein SLEEPY1 upon GA binding, leading to RGL2 ubiquitination and subsequent degradation during seed germination (Ariizumi et al. 2011; Hirano et al. 2008; Xu et al. 2021). Ariizumi demonstrated that *sleepy1* mutants accumulate elevated RGL2 protein levels in both dormant and after-ripened seeds (Ariizumi and Steber 2007; Strader et al. 2004). They postulate that accumulated RGL2 in after ripened *sleepy1* seeds may be functionally inactive or subject to regulation by additional factors, and RGL2 degradation is not strictly required for germination completion (Ariizumi and Steber 2007). ABA signaling intersects with RGL2-mediated regulation of dormancy and germination: stabilized RGL2 enhances endogenous ABA levels by upregulating *XERICO* expression, which promotes ABA accumulation through undefined mechanisms, while ABA exposure during imbibition elevates RGL2 transcription (Hu et al. 2019; Piskurewicz et al. 2008). GA, one of the primary phytohormones, coordinate with ABA to regulate seed dormancy and germination, with RGL2 serving as the central regulatory component in this process.

ABA serves as a critical phytohormone mediating diverse physiological processes, including seed maturation, growth regulation, and developmental regulation, and adaptive responses to environmental stresses (Jia et al. 2022; Kavi Kishor et al. 2022; Krüger et al. 2025; Liu et al. 2022; Wu et al. 2024; Zhang et al. 2021). ABA is perceived by PYR/PYL/RCAR receptors, which inhibit PP2C phosphatase activity to activate the SnRK2 kinase cascade. This pathway enables rapid modulation of downstream transcription factors (ABIs, ABFs/AREBs) and ion channels while integrating environmental cues such as osmotic stress, temperature, and light. These integrated signals mediate critical physiological responses including seed dormancy regulation, stomatal closure, and stress tolerance (Ali et al. 2020; Hsu et al. 2021; Li et al. 2023; Waadt et al. 2022). ABA acts as a positive regulator during seed maturation, where it promotes dormancy induction, while also maintaining dormancy in imbibed seeds through the PYR/PYL/RCAR receptor-mediated signaling pathway (De Brasi-Velasco et al. 2023; Wang et al. 2024b). Dormancy is reduced in ABA-deficient seeds generated by mutations, chemical inhibition of ABA biosynthesis, or in ABA-insensitive mutants, whereas overexpression of ABA biosynthetic enzyme genes and decreasing ABA degradation led to enhanced dormancy (Ali et al. 2022; Finkelstein et al. 2002; Hao et al. 2024; Himmelbach et al. 2003). ABA signaling integrates temperature cues to regulate dormancy cycling and germination potential (Otani et al. 2024; Sacharowski et al. 2025; Xu et al. 2022). The ABA-Insensitive (ABI) family in *Arabidopsis thaliana* serves as key transcriptional regulators within the ABA signaling pathway, comprising three canonical members: ABI3, ABI4, and ABI5 (named for their reduced ABA sensitivity in corresponding mutants). These factors play pivotal roles in mediating seed development, dormancy, germination, and stress responses (Cheng et al. 2025; Finkelstein et al. 2022; Xiao et al. 2017). ABI5 functions as a central transcriptional regulator in seed germination control, serving as the master inhibitory node that integrates ABA and GA, and light signaling pathways into the regulatory network governing seed germination (Hu et al. 2019; Ling et al. 2017; Piskurewicz et al. 2008; Zhao et al. 2022). Furthermore, ABI5 exhibits functional associations with amylase genes (Fan et al. 2025; Huang et al. 2021; Li et al. 2022a, b; Song et al. 2022). However, the precise role of ABA in stratification-mediated dormancy release requires further investigation, particularly regarding its interaction with ABI5-dependent signaling pathways.

The antagonistic regulatory roles of GA and ABA in seed germination have been extensively characterized across diverse plant species (Fu et al. 2025; Achard et al. 2006; Oh et al. 2007; Seo et al. 2009). Multiple regulatory nodes mediating ABA-GA crosstalk have been identified (Li et al. 2022a, b; Shu et al. 2016; Xian et al. 2024a, b). FUS3 serves as a critical regulatory node in ABA-GA crosstalk, with its Arabidopsis ortholog mediating positive regulation of ABA biosynthesis and negative regulation of GA biosynthesis pathways (Buhrow et al. 2021; Tsai and Gazzarrini 2012). DAG1 functions as a negative regulator of seed germination in *Arabidopsis thaliana*, directly repressing the expression of the GA biosynthesis gene *GA3ox1* and the ABA catabolic gene *CYP707A2* (Boccaccini et al. 2016; Gabriele et al. 2010). Notably, RGL2 functions as the primary DELLA protein inhibiting seed germination, acting through physical interactions with ABI4 and NF-YC3/NF-YC4/NF-YC9 subunits as a transcriptional coactivator by enhancing expression of the ABA-responsive transcription factor ABI5, thereby amplifying ABA signaling pathways (Alabadí and Sun 2025; Liu et al. 2016; Xian et al. 2024a, b). The amounts of both RGL2 and ABI5 proteins are positively regulated by ABA and negatively regulated by GA (Kazachkova et al. 2016; Xian et al. 2024a, b). Plant hormones typically regulate seed germination by modulating α-amylase activity and starch mobilization. α-amylase activity is positively correlated with germination rate. Salinity stress and chilling stress reduces GA levels while increasing ABA levels, thereby inhibiting α-amylase activity and consequently suppressing starch hydrolysis, which ultimately impedes germination (Wang et al. 2024a; Chen et al. 2025; Ikram et al. 2025; Lu et al. 2025; Ma et al. 2022; Nie et al. 2022). The precise regulation of seed dormancy and germination relies on the dynamic balance and complex interactions between germination-promoting GA and dormancy-maintaining ABA. The molecular basis lies in the intricate interplay between the two signaling pathways at multiple levels (Liao et al. 2023). Furthermore, cold stratification is an effective dormancy-breaking treatment and reduces ABA content while increasing GA levels, accompanied by enhanced enzyme activities and shifts in storage substances (Chen et al. 2025). Studying the antagonistic relationship between GA and ABA is of great significance for revealing the molecular switch controlling seed dormancy release and germination initiation, optimizing seed germination regulation in agricultural production, and improving crop stress tolerance.

The transition between dormancy and germination represents a critical phase in the life cycle of higher plants, where physiological dormancy is regulated by dormancy-regulating factors such as after-ripening, stratification, and ABA/GA signaling. In *Arabidopsis thaliana*, seed dormancy is alleviated through moist chilling (stratification) or after-ripening processes (Holdsworth et al. 2008; Lee et al. 2021Pausas et al. 2022). This study aims to elucidate how stratification coordinates hormonal crosstalk between ABA and GA signaling pathways to regulate starch hydrolysis during seed dormancy release. Using a panel of well-characterized mutants including the GA biosynthesis-deficient mutant *ga3ox1*, GA-insensitive mutant *sleepy1*, ABA catabolism-impaired mutant *cyp707a2*, and the double mutant *cyp707a2 sleepy1*, we analyzed the hormonal responses of dormancy alleviation under stratification. The results demonstrated that stratification significantly enhanced GA biosynthesis and relieved RGL2-mediated repression of α-amylase gene expression. Furthermore, stratification alleviated ABA-dependent suppression of starch hydrolysis during germination. Notably, both RGL2 and ABI5 were found to promote starch hydrolysis, providing new insights into the hierarchical regulation of dormancy release by hormonal signaling networks.

## Results

### Cold-pretreatment decreased the inhibition of ABA on seed germination

**Fig. 1:**
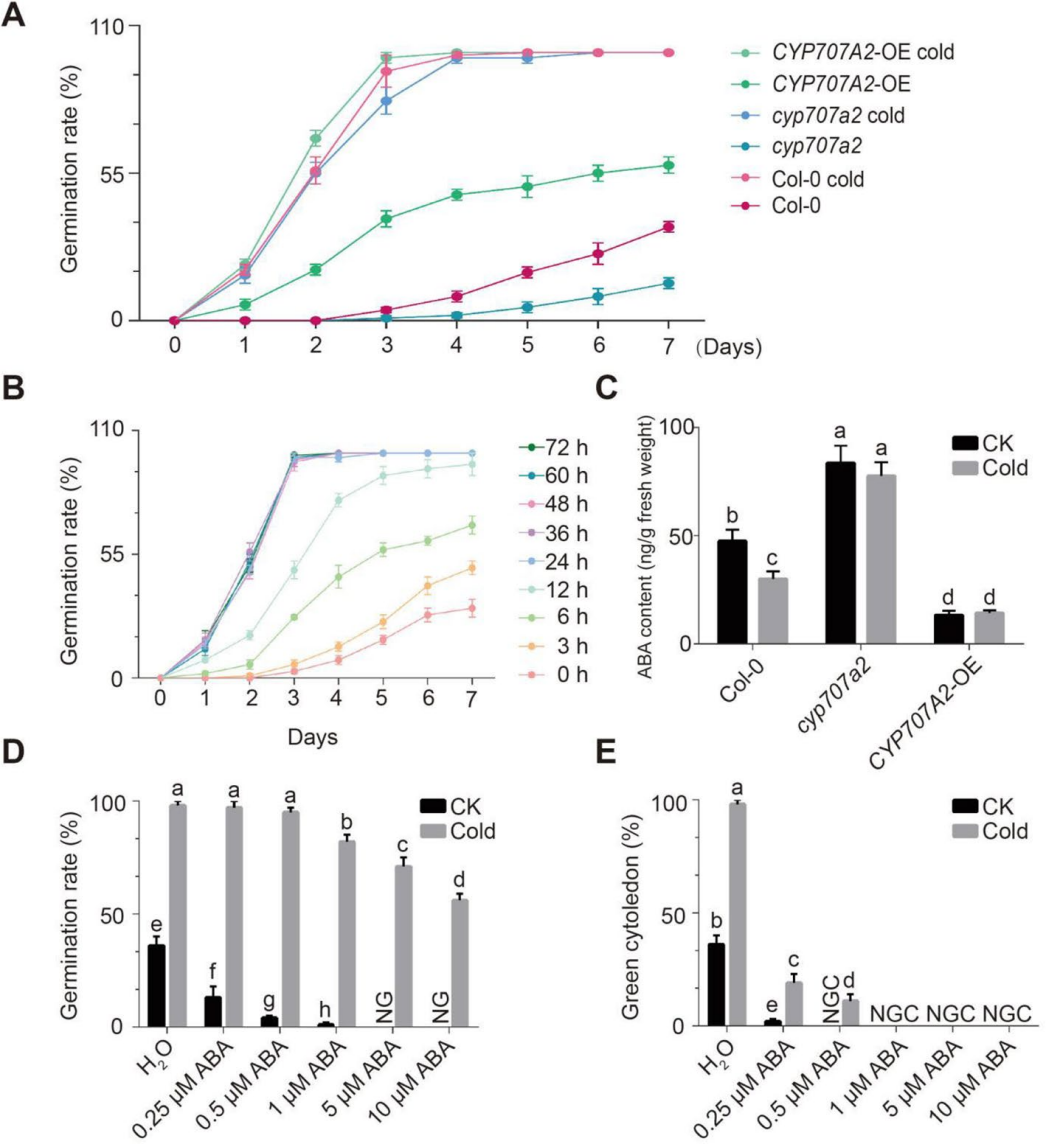
The effect of stratification and different concentrations of ABA on seed dormancy break. **A** The effect of stratification on seed dormancy break of freshly harvested mutant seeds. The seeds underwent a 24-h stratification treatment before being transferred to normal conditions for germination testing. **B** The effect of different stratification time on seed dormancy break of freshly harvested WT seeds. The seeds underwent stratification pre-treatment for varying durations, and the seed germination rate was recorded within 7 days. **C** The effect of stratification on ABA content. The ABA contents were accounted after 24 h imbibition with or without stratification in wild type and different mutant seeds. **D** The effect of stratification and different concentrations of ABA on seed germination after 7 days imbibition. **E** The effect of stratification and different concentrations of ABA on green cotyledon emergence after 7 days imbibition. Seeds were treated with (Cold) or without stratification (CK). NG: No germination. NGC: No green cotyledon. Different letters above columns indicate significant differences (one-way ANOVA, *P* < 0.05)

Our previous research showed that *CYP707A2* mediates ABA catabolism during seed germination and *cyp707a2* mutant exhibits strong dormancy when imbibed with water under 21℃ (Liu et al. 2009). Here we found that the seed dormancy of freshly harvested *cyp707a2* was effectively broken by cold-pretreatments (stratification) (Fig. 1A). Stratification also broke the dormancy of freshly harvested wild-type (WT) seeds completely after the treatment for more than 24 h (Fig. 1B). ABA content is crucial for breaking seed dormancy (Merino et al. 2025). Stratification decreased the ABA contents in WT but did not change the ABA contents in *cyp707a2* mutant and *CYP707A2-OE* after 24 h imbibition (Fig. 1C). The expressions of ABA biosynthesis and catabolism genes were also changed under stratification (Fig. S1). Results indicated that stratification increased the expressions of ABA biosynthesis gene *NCED6* and ABA catabolism genes *CYP707A1* and *CYP707A2* at the first imbibition period.

The effect of stratification on ABA inhibition on seed germination was also investigated. With the increase of ABA concentrations, seed germination could be inhibited completely. Such inhibition was reversed by stratification (Fig. 1D). Stratification also slightly reversed the inhibition exerted by low concentration of ABA on green cotyledon emergence, but the inhibition by higher ABA concentration could not be reversed (Fig. 1E). In addition, we collected seedlings under CK and Cold conditions with ABA treatment at the initial imbibition stage (D0), radicle emergence stage (D2), and green cotyledon emergence stage (D5), and measured α-amylase activity and the expression levels of related *AMY* genes. The results showed that α-amylase activity peaked at the radicle emergence stage (D2), with activity in the Cold treatment being substantially higher than that in the CK group. The expression levels of *AMY1* and *AMY3* increased progressively with germination time, whereas *AMY2* expression was most prominent at D0. Notably, across all time points, gene expression levels in the Cold treatment were consistently and markedly higher than those in the CK group (Fig. S2). The results indicated that stratification could break the inhibitory level of ABA on seed germination.

### GA biosynthesis was essential in stratification-induced seed dormancy breaking

**Fig. 2:**
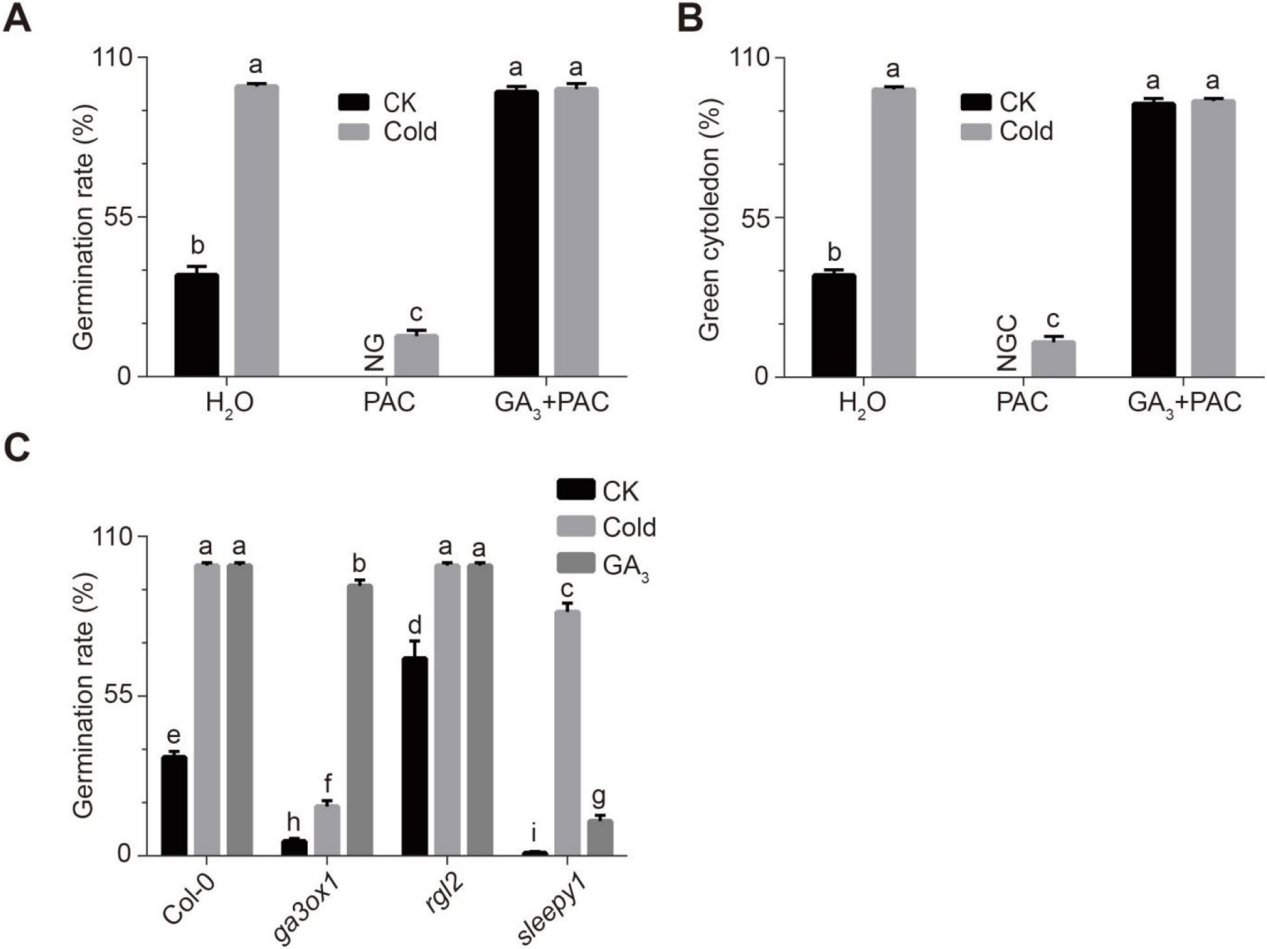
The effect of stratification and GA on seed dormancy break in WT and different mutant seeds. **A** The effect of stratification, GA and its synthesis inhibitor PAC on seed germination. **B** The effect of stratification, GA and its synthesis inhibitor PAC on green cotyledon emergence. **C** The effect of stratification and GA on seed germination in WT and different mutant seeds. Seeds were treated with (Cold) or without stratification (CK). NG: No germination. NGC: No green cotyledon. Different letters above columns indicate significant differences (one-way ANOVA, *P* < 0.05)

It has been known that stratification triggers GA biosynthesis and promotes seed germination (Yamauchi et al. 2004). Our results showed that stratification could break the dormancy of freshly harvested seeds but would not have effect if GA biosynthesis was inhibited by PAC. Exogenous GA reversed the function of PAC completely (Fig. 2A). Similar results were obtained with the green cotyledon emergence (Fig. 2B).

We also measured the expressions of GA biosynthesis genes and response genes during germination with or without cold treatments. As expected, both GA biosynthesis genes and response genes were induced by stratifications (Fig. S3). To investigate the role of GA, we used some mutants, including *ga3ox1,* which has decreased GA biosynthesis under normal condition and stratification (Yamauchi et al. 2004); *rgl2,* which can confer the germination ratio corresponding to wild type (WT) on the GA deficiency mutant (Penfield et al. 2006); and *sleepy1,* which accumulates high level of RGL2 and blocks the signaling pathway of GA (Piskurewicz et al. 2008). The *rgl2* mutant exhibited less dormancy in freshly harvested seed. Stratification treatment broke seed dormancy of WT, *rgl2* and *sleepy1*, but could not break the dormancy of *ga3ox1* (Fig. 2C). While exogenous GA treatment broke the seed dormancy of WT, *ga3ox1* and *rgl2*, it could not break the seed dormancy of *sleepy1* when compared with others (Fig. 2C). In addition, the *rgl2* mutants exhibited higher GA content, and stratification treatment significantly increased GA content in both WT and *rgl2* mutants (Fig. S4). These results indicated that GA biosynthesis is essential for the stratification to break dormancy and the degradation of RGL2 was needed in the GA-induced seed germination. It was possible to speculate that stratification blocks the inhibition of RGL2 on seed germination.

### ABA and GA regulated α-amylase activity under cold-pretreatment

**Fig. 3:**
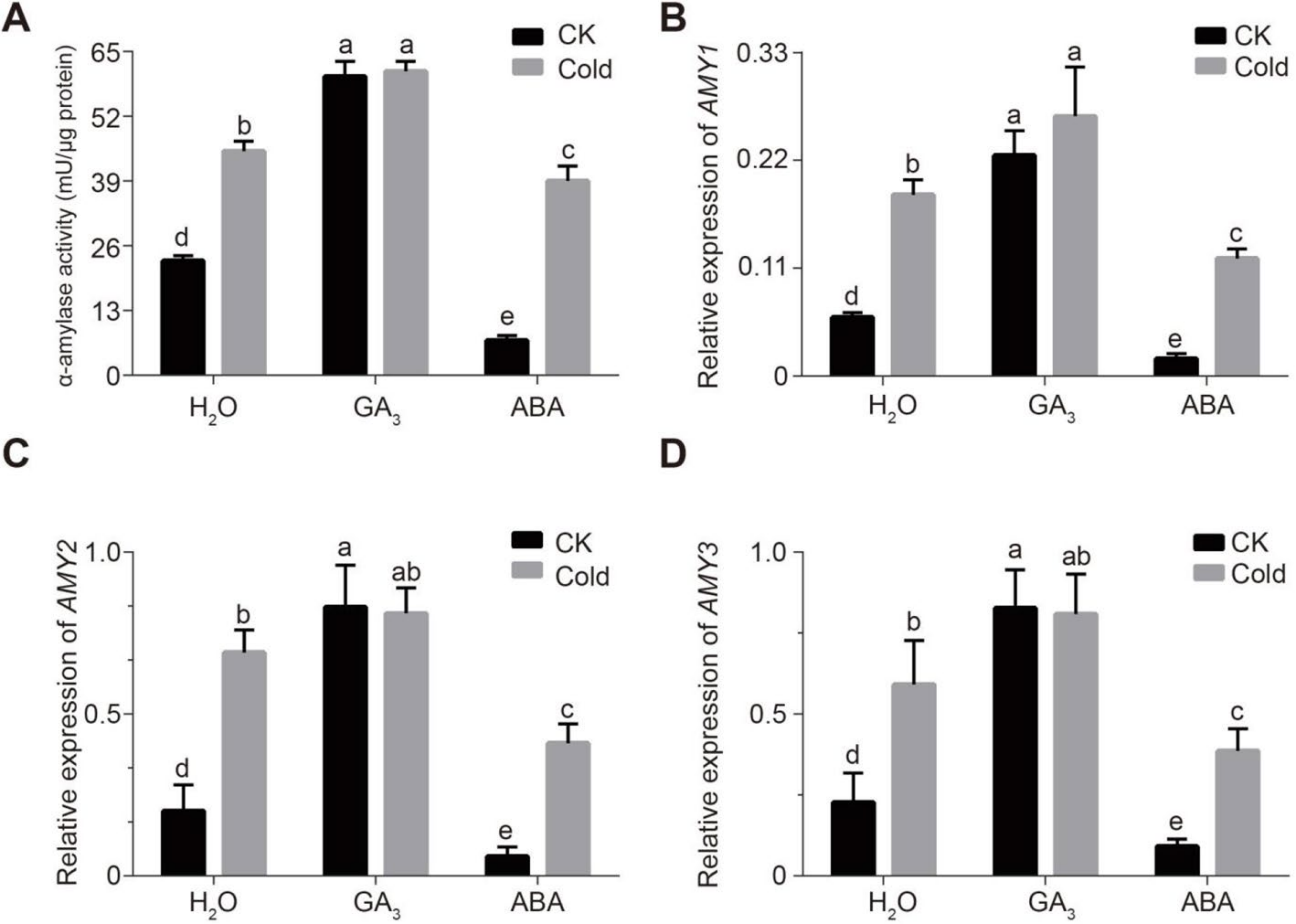
The effect of stratification, ABA and GA on α-amylase activity and their genes expression. **A** The effect of stratification, ABA and GA on the activity of α-amylase. **B** The effect of stratification, ABA and GA on the expression of *AMY1*. **C** The effect of stratification, ABA and GA on the expression of *AMY2*. **D** The effect of stratification, ABA and GA on the expression of *AMY3*. Seeds were treated with (Cold) or without stratification (CK). The activity of α-amylase and the genes expression were analyzed after 24 h imbibition with or without stratification. Different letters above columns indicate significant differences (one-way ANOVA, *P* < 0.05)

Α-amylase is a pivotal enzyme in starch hydrolysis in plant (Aghaei et al. 2022), it was induced by GA in aleurone during seed germination and initiates the degradation of starch granules in the non-living endosperm (Bethke et al. 2007). If the GA biosynthesis genes were mutant or GA biosynthesis was inhibited by PAC, the seeds would not germinate (Toh et al. 2008). So, the activity of α**-**amylase was selected as the marker of starch hydrolysis and seed germination in this study. The results showed that ABA inhibited the activity of α-amylase. Stratification significantly relieved the inhibitory effect of ABA on α-amylase activity (Fig. 3A). Previous research indicated that GA_3_ promotes the expression of α-amylase in barley and rice, while ABA has the reversed effect (Chen et al. 2006; Loreti et al. 2000). GA_3_ promoted the activity of α-amylase with the same state of stratification (Fig. 3A). The expression of α-amylase gene also showed a corresponding trend of change. Especially, the inhibition by ABA could be overridden by stratifications (Fig. 3B-D).

**Fig. 4:**
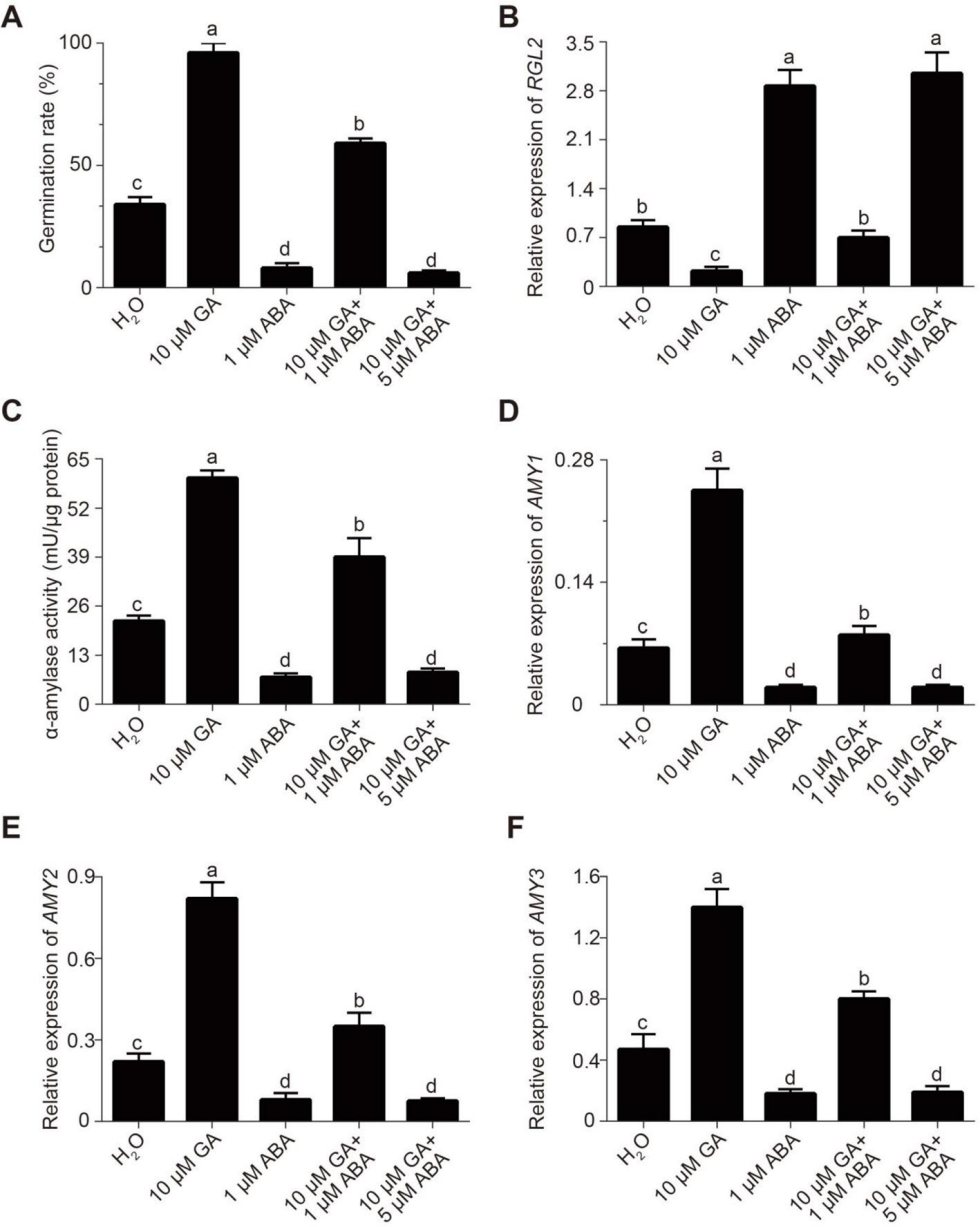
The relationship of ABA and GA in seed germination and gene expression. **A** The effect of different concentrations of ABA and GA on seed germination. **B** The effect of different concentrations of ABA and GA on the expression of *RGL2*. **C** The effect of different concentrations of ABA and GA on the activity of α-amylase. **D** The effect of stratification, ABA and GA on the expression of *AMY1*. **E** The effect of stratification, ABA and GA on the expression of *AMY2*. **F** The effect of stratification, ABA and GA on the expression of *AMY3*. Different letters above columns indicate significant differences (one-way ANOVA, *P* < 0.05)

Antagonistic roles of ABA and GA have been well documented in seeds of many plant species (Binenbaum et al. 2023; Xian et al. 2024a, b). To investigate the antagonistic roles of GA and ABA on α-amylases, different concentrations of ABA and GA were applied. High concentration (10 μM) of GA could reverse the inhibition of seed germination caused by low concentration (1 μM) ABA, but not that caused by high concentration (5 μM) ABA. There exists a dose-dependent antagonistic interaction between ABA and GA in controlling germination (Fig. 4A). ABA treatment enhanced the expression of *RGL2,* conversely, GA treatment decreased the expression of *RGL2*. Also, the enhanced expression of *RGL2* caused by low concentration of ABA could be reversed by GA (Fig. 4B). In addition, the activity of α-amylase was also inhibited by ABA and induced by GA. Similarly, the inhibition effect was caused by low concentration of ABA, not its high concentration, and it can be reversed by high concentration of GA substantially (Fig. 4C). The similar results were also obtained from the expression analysis of the three α-amylase genes. It should be noted that the expression of *AMY3* was much higher than those of the other two genes (Fig. 4D-F). We analyzed *amy1*, *amy2*, and *amy3* single mutants under GA₃, ABA, and GA₃ + ABA conditions with or without stratification. Under GA₃ treatment alone, all genotypes germinated efficiently at 21 °C, and stratification further raised germination to nearly 100% in all lines, indicating that GA effectively activates α‑amylase redundantly. Under ABA treatment, the single mutants were more sensitive to ABA, and the differential changes after stratification may be attributed to gene redundancy, with AMY1 and AMY3 potentially compensating more, leading to a higher germination rate in *amy2*. When GA₃ was added together with ABA, stratification again improved germination, with *amy2* exhibiting the largest cold‑induced increase, confirming that α-amylase is a key downstream target for ABA and GA antagonistic regulation, and stratification can "unlock" this target when ABA dominates, reflecting the reprogramming of hormone signaling networks by environmental signals (Fig. S5). The results showed that ABA and GA have antagonistic effects on the activity and gene expression of α-amylase during seed germination, and the inhibitory effect of ABA could be alleviated in stratification. At the same time, the expression of *RGL2* was also regulated by ABA and GA and was associated with changes in the activity and gene expression of α-amylase.

### RGL2 regulated α-amylase activity under GA and ABA conditions

**Fig. 5:**
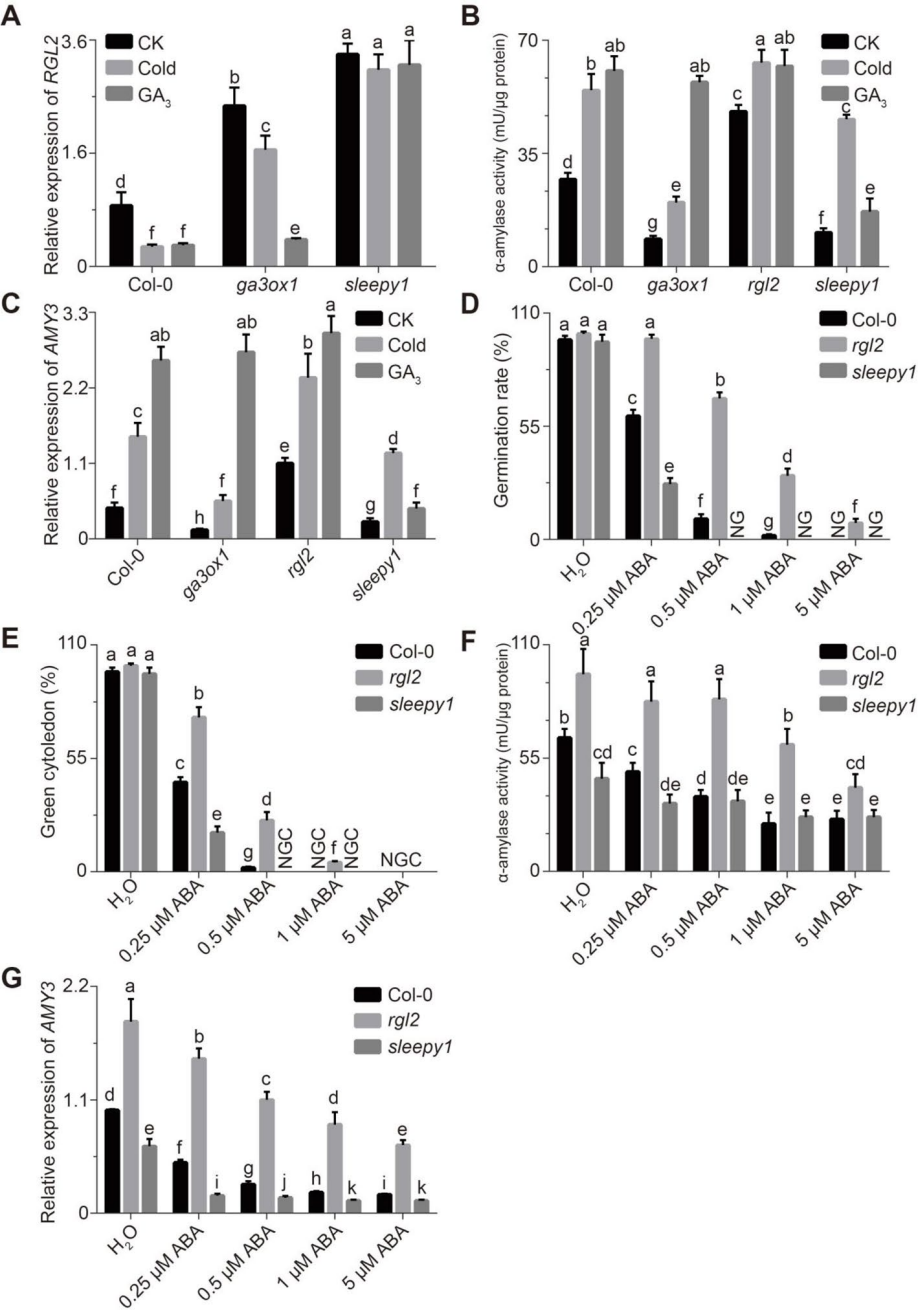
The role of *RGL2* in ABA-regulated α-amylase activity. **A** Stratification and GA treatment affected the expression of *RGL2* in WT and different mutants. **B** Stratification and GA treatment affected the activity of α-amylase in WT and different mutants. **C** Stratification and GA treatment affected the expression of *AMY3* in WT and different mutants. **D** The effect of different concentrations of ABA on seed germination in WT, *rgl2* and *sleepy1*. **E** The effect of different concentrations of ABA on green cotyledon emergence in WT, *rgl2* and *sleepy1*. **F** The effect of different concentrations of ABA on the activity of α-amylase in WT, r*rgl2* and *sleepy1*. **G** The effect of different concentrations of ABA on the expression of *AMY3* in WT, *rgl2* and *sleepy1*. Seeds were treated with (Cold) or without stratification (CK) for 24 h. The activity of α-amylase and the genes expression were analyzed after 24 h imbibition with or without stratification. The genes expression were analyzed after 24 h imbibition under different concentrations of ABA. NG: No germination. NGC: No green cotyledon. Different letters above columns indicate significant differences (one-way ANOVA, *P* < 0.05)

Previous research suggested that RGL2 played an important role in GA regulated seed dormancy (Gao et al. 2021; Mérai et al. 2024). In this study, *ga3ox1*, *sleepy1*, *rgl2* mutants were used to investigate the role of RGL2 on GA and cold-regulated α-amylase activity. The expression of *RGL2* was determined with or without stratifications and GA treatments, *ga3ox1* and *sleepy1* accumulated high levels of *RGL2* mRNA. Stratifications decreased the expression of *RGL2* in WT markedly (by 66%) and slightly in *ga3ox1* and *sleepy1* mutants (only 28.9% and 11.8% respectively). GA_3_ treatment decreased *RGL2* expression in WT and *ga3ox1* obviously, but slightly in *sleepy1* mutants (Fig. 5A). The activity of α-amylase and the expression of *AMY3* were also analyzed. Results indicate that stratifications increased the activity of α-amylase in WT and *sleepy1* mutants substantially, but only slightly in *ga3ox1* mutant (Fig. 5B). GA treatment increased the activity of α-amylase in WT and *ga3ox1* mutants obviously and slightly in *sleepy 1* mutant (Fig. 5B). The similar effects caused by stratification and GA treatment were also obtained from the expression analysis of *AMY3* (Fig. 5C). While the activity of α-amylase and the expression of *AMY3* were maintained at a higher level under all the conditions.

Piskurewicz et al. (2008) reported that RGL2 stimulates ABA synthesis in ABA regulated seed germination. In this study, we used *sleepy1*and *rgl2* to look at its effect. Results indicate that with the increase of ABA concentration, the germination ratio of non-dormancy seeds decreased. With the treatment of 0.5 µM ABA, the germination ratio decreased to less than 20%, when with the treatment of 5 µM ABA, the germination ratio decreased to zero in WT (Fig. 5D). *rgl2* mutant was much insensitive to ABA compared with WT. The germination ratio of *rgl2* mutant maintained at 68% under the treatment of 0.5 µM ABA, and even under the treatment of 5 µM ABA, it still maintained at 8%. As a comparison, the *sleepy1* mutant was much more sensitive to ABA, and 0.5 µM ABA blocked the seed germination completely (Fig. 5E). The emergence of green cotyledon was also measured under the same condition. Results indicate that insensitivity of *rgl2* to ABA was decreased. Under the treatment of 0.5 µM ABA, the germination ratio of this mutant maintained at 68%, while only 25% of them exhibited green cotyledon after 7 days inhibition (Fig. 5D-E).

The activity of α-amylase and the expression of *AMY3* were also determined. Results indicate that ABA treatment decreased the activity of α-amylase and the expression of *AMY3*. *rgl2* mutant showed insensitivity, while *sleepy1* showed sensitivity under the treatment with the same ABA concentration (Fig. 5F-G). *RGL2* plays an important role in seed dormancy and α-amylase activity regulated by GA and ABA. Stratification and GA treatment can reduce *RGL2* expression and increase α-amylase activity and *AMY3* expression, while ABA treatment is the opposite.

### Cold-pretreatment blocked the regulation of RGL2 on α-amylase

**Fig. 6:**
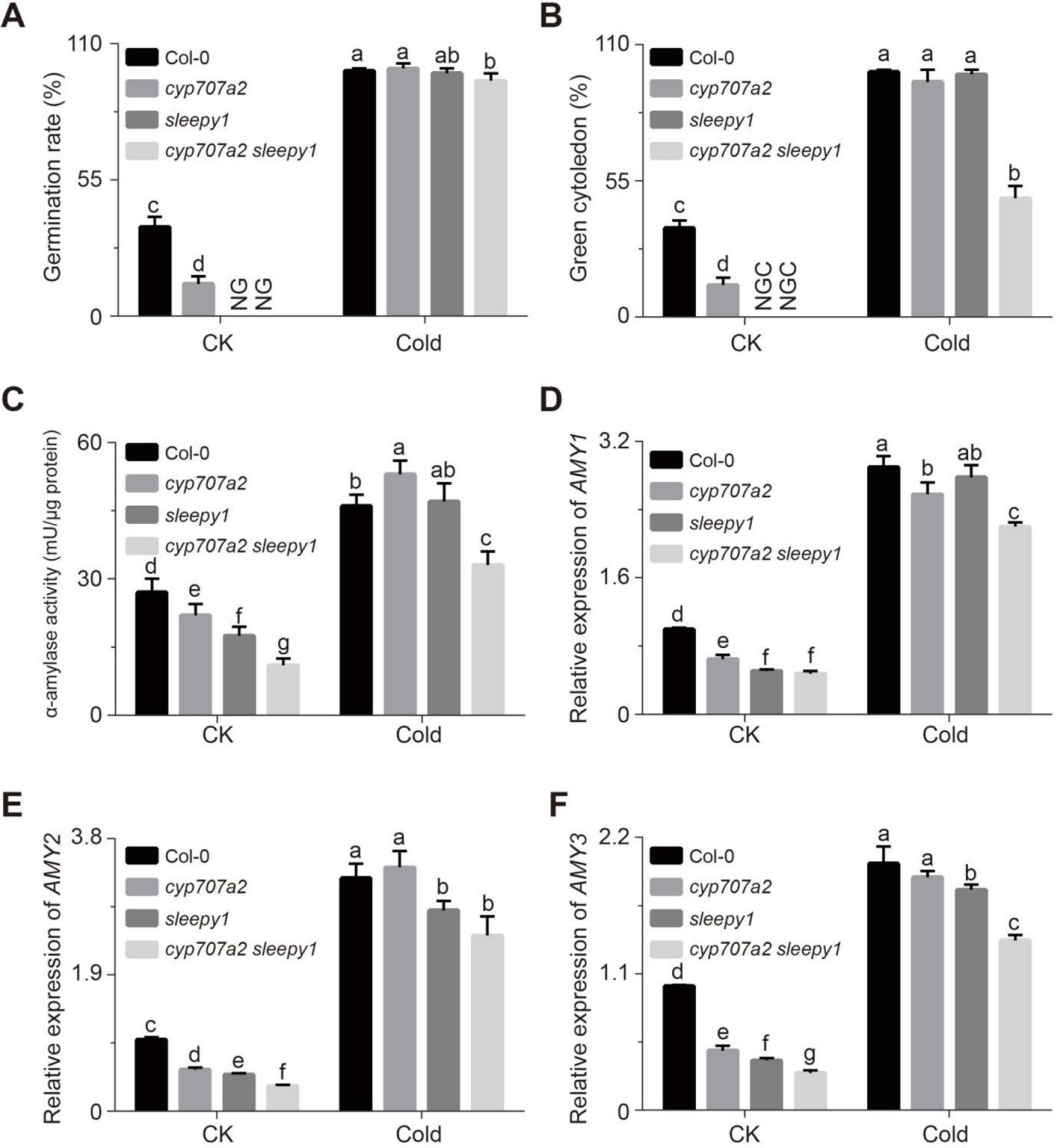
Phenotypic and gene expression analysis of the WT, *cyp707a2*, *sleepy1* and *cyp707a2 sleepy1* double mutant. **A** Germination of freshly harvested seeds in WT and different mutants. **B** Green cotyledon emergence of freshly harvested seeds in WT and different mutants. **C** The effect of stratification on the activity of α-amylase in WT and different mutants. **D** The effect of stratification on the expression of *AMY1* in WT and different mutants. **E** The effect of stratification on the expression of *AMY2* in WT and different mutants. **F** The effect of stratification on the expression of *AMY3* in WT and different mutants. Seeds were treated with (Cold) or without stratification (CK) for 24 h. The activity of α-amylase and the genes expression were analyzed after 24 h imbibition with or without stratification. NG: No germination. NGC: No green cotyledon. Different letters above columns indicate significant differences (one-way ANOVA, *P* < 0.05)

To further elucidate the function of RGL2 on ABA and cold-regulated dormancy break, *cyp707a2*, *sleepy1* and their double mutant *cyp707a2 sleepy1* were selected and constructed (Fig. S6). Freshly harvested *cyp707a2*、*sleepy1* and *cyp707a2 sleepy1* had strong dormancy compared with WT, and stratification broke the dormancy of WT、*cyp707a2*、*sleepy1* and their double mutant *cyp707a2 sleepy1* completely (Fig. 6A). When the emergence of green cotyledon is concerned, stratification reversed the inhibition of WT, *cyp707a2* and *sleepy1* freshly harvest seeds, but could not reverse the inhibition of double mutant *cyp707a2 sleepy1* on green cotyledon emergence completely (Fig. 6B). This result indicated that stratification could completely break the seed dormancy of WT and the mutants.

The activity of α-amylase and the expressions of *AMYs* were also analyzed. α-amylase was slightly decreased in *cyp707a2* mutant and substantially decreased in *sleepy1* and their double mutant *cyp707a2 sleepy1* after 24 h imbibition. Stratification increased the activity of α-amylase in both WT and the three mutants (Fig. 6C). The expressions of *AMYs* were analyzed under the same condition, results indicated that the expression of all three genes were slightly decreased in *cyp707a2* and sharply decreased in *sleepy1* and the double mutant *cyp707a2 sleepy1*. Conversely, stratification increased their expressions (Fig. 6D-F). This result indicated that stratification could enhance the activity of α-amylase and the expression of *AMYs* genes in WT and the mutants.

**Fig. 7:**
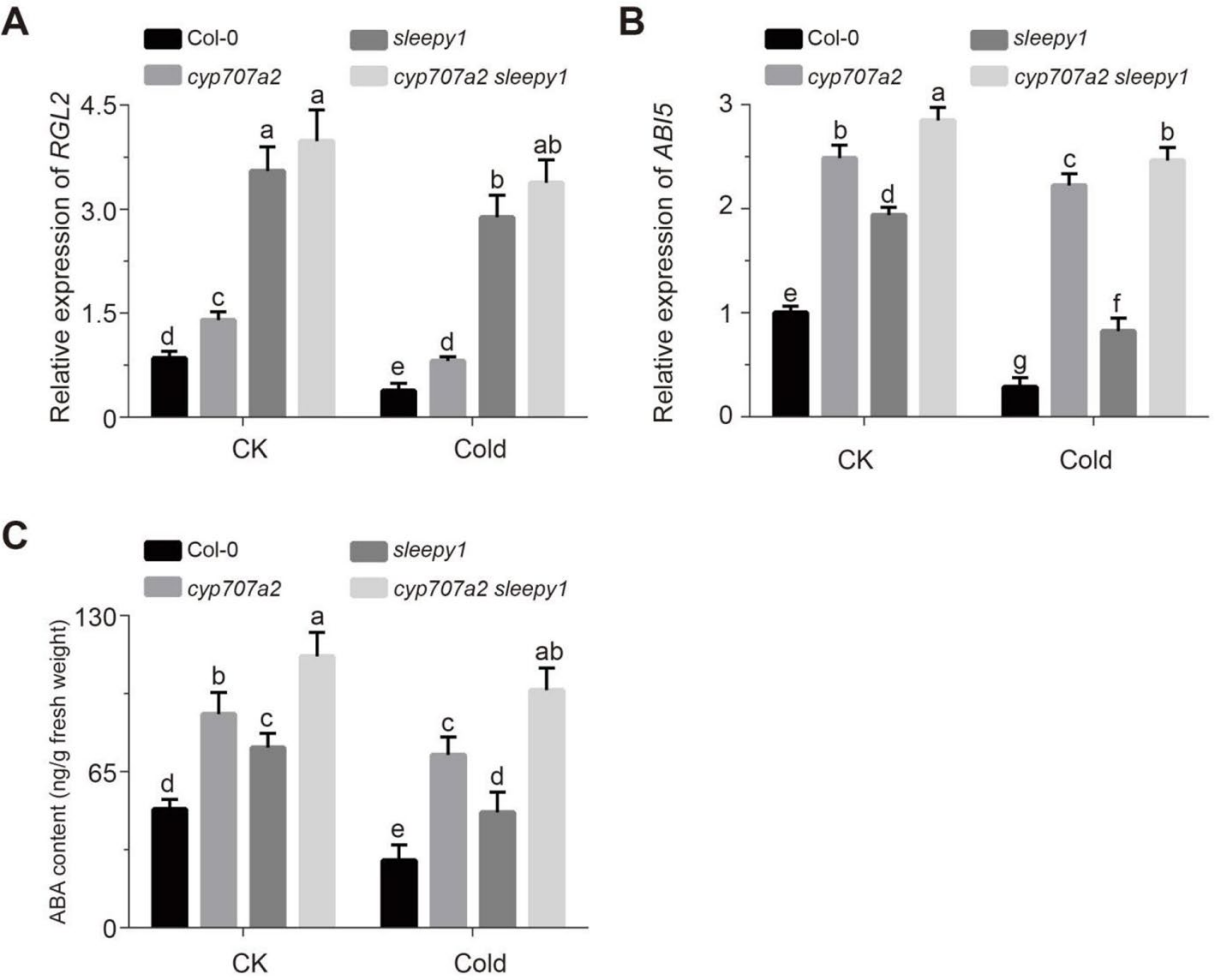
The effect of stratification on the gene expression and ABA content in WT and different mutants. **A** The effect of stratification on the expression of *RGL2* in WT and different mutants. **B** The effect of stratification on the expression of *ABI5* in WT and different mutants. **C** The effect of stratification on ABA content in WT and different mutants. Seeds were treated with (Cold) or without stratification (CK) for 24 h. The gene expressions and ABA content were analyzed after 24 h imbibition with or without stratification. Different letters above columns indicate significant differences (one-way ANOVA, *P* < 0.05)

The expressions of *RGL2* and *ABI5* were also compared under same condition (Fig. 7). Results indicated that *sleepy1* and their double mutant *cyp707a2 sleepy1* exhibited much higher expression of *RGL2* than WT and *cyp707a2* mutant after 24 h imbibition at 21 ℃. Stratifications decreased the expression of *RGL2* substantially in WT and *cyp707a2* mutant (55.3% and 42% respectively), while only slightly in *sleepy1* and double mutant *cyp707a2 sleepy1* (19.1% and 15.5% separately) (Fig. 7A). The expression of *ABI5* was different from *RGL2.* The expression of *ABI5* was much higher in *cyp707a2*, *sleepy1* and double mutant *cyp707a2 sleepy1* than in WT. Stratifications decreased the expression of *ABI5* in WT and *sleepy1*, but not in *cyp707a2* and the double mutant *cyp707a2/sleepy1* after 24 h imbibition (Fig. 7B). The ABA content in *cyp707a2*, *sleepy1* and double mutant *cyp707a2 sleepy1* was higher than WT after 24 h imbibition. And stratifications decreased ABA content in WT and *sleepy1*, but not in *cyp707a* and double mutant *cyp707a2 sleepy1* (Fig. 7C). Stratification broke the dormancy of both WT and mutant seeds by reducing ABA content and *RGL2* expression, thereby enhancing α-amylase activity and *AMYs* gene expression.

## Discussion

Dormancy is a trait of considerable agronomic and ecological importance, which enhances plant fitness by protecting the embryo and preventing out-of-season germination (Krüger et al. 2025; Nakabayashi et al. 2021; Zhang et al. 2022). Freshly harvested *Arabidopsis thaliana* seeds typically exhibit strong dormancy. Previous studies have indicated that seed dormancy is regulated by plant hormones, small signaling compounds, and environmental factors (Dorone et al. 2021; Hubert et al. 2024; Regnard et al. 2024). Dormancy can also be broken via an after-ripening period or stratification. In this study, our observations show that GA biosynthesis, but not GA signaling, is mediated by RGL2, which is essential for stratification-induced seed dormancy breaking (Fig. 2). The GA-triggered expression and activity of α-amylase are blocked in the *sleepy1* mutant, but this effect can be reversed by stratification (Fig. 5). ABA strongly inhibits seed germination, and this inhibition is completely reversed by stratification. However, the inhibition of seedling development by ABA cannot be reversed by stratification (Fig. 1). The expression and activity of α-amylase are inhibited by ABA via the RGL2-mediated pathway, but stratification also blocks the inhibition of ABA on α-amylase (Figs. 5, 6 and 7). RGL2 has been reported as a node of crosstalk between ABA and GA in the regulation of seed germination (Xian et al. 2024a, b). Our results also show that the regulation of ABA and GA via RGL2 is blocked by stratification.

Stratification is widely used to break seed dormancy and improve the frequency of germination (Cheng et al. 2022; Hao et al. 2024). Our results show that, under moist conditions, 24 h of stratification can completely break the dormancy of freshly harvested *Arabidopsis thaliana* seeds (Col-0) (Fig. 1). Our previous results indicated that *cyp707a2* mutant seeds accumulate ABA after imbibition and exhibit strong dormancy (Liu et al. 2009). In this study, we found that, although *cyp707a2* mutant seeds accumulate higher levels of ABA under both 21℃ and stratification, their dormancy is broken by stratification. When higher exogenous ABA (5 µM) was applied, seed germination was completely inhibited under 21℃, but stratification overrode this inhibitory effect. However, even after stratification, the emergence of green cotyledons was still inhibited by ABA (Fig. 1). These results indicate that stratification blocks the inhibitory effect of ABA on seed germination but has little impact on seedling development.This mechanism deepens our understanding of hormonal co-regulation. It demonstrates that environmental signals exert their influence not merely by altering absolute hormone levels, but more importantly by reshaping the internal connectivity of signaling pathways. From a physiological perspective, seed germination primarily relies on the mobilization of stored reserves in the endosperm (such as starch hydrolysis) and radicle cell elongation for emergence, whereas cotyledon greening involves more complex processes including plastid differentiation, chlorophyll synthesis, and the establishment of photosynthetic apparatus. Stratification overcomes ABA-mediated germination inhibition by sufficiently restoring the energy supply required for starch hydrolysis and radicle elongation through releasing RGL2/ABI5-imposed repression on α-amylase expression. However, ABA-induced cotyledon greening inhibition may involve more extensive or persistent physiological barriers, such as suppression of key genes for chloroplast development, interference with photosynthetic system assembly, or inhibition of cell division/expansion. These processes may not fully depend on α-amylase-mediated starch hydrolysis and cannot be reversed by short-term stratification. Multiple checkpoints exist between seed dormancy release and seedling establishment, and future studies could further dissect specific ABA signaling modules controlling cotyledon development.

GA acts as an antagonistic role to ABA in seed germination in many plant species (Binenbaum et al. 2023). Does stratification reverse the inhibition of ABA on seed germination through the antagonistic action of GA? It has been reported that stratification triggers GA biosynthesis to promote seed germination (Yamauchi et al. 2004). We tested this hypothesis using PAC, an inhibitor of GA biosynthesis. Results showed that seeds treated with PAC maintained dormancy even after stratification (Fig. 2), indicating that GA biosynthesis is required for the effect of stratification. PAC treatment also inhibited seed germination at the normal growth temperature of *Arabidopsis thaliana*.

RGL2 is a conserved repressor of GA signaling that acts immediately downstream of the GA receptor to modulate seed development in *Arabidopsis thaliana* (Yang et al. 2020a, b). RGL2 has been reported to be responsive to GA-induced degradation via the ubiquitin–proteasome pathway (Lee et al. 2022; Ponnu 2022). Here, we explored its relevance to stratification using three mutants *rgl2*, *sleepy1*, and *ga3ox1*. The *rgl2* mutant, which lacks RGL2, exhibits a germination rate comparable to that of the wild type under GA deficiency (Penfield et al. 2006; Zentella et al. 2007). The *sleepy1* mutant, which accumulates higher levels of RGL2, can block the GA signaling pathway (Piskurewicz et al. 2008). The *ga3ox1* mutant, which has decreased GA biosynthesis under normal conditions or stratification, is required for cold-stimulated seed germination (Yamauchi et al. 2004). Stratification broke seed dormancy of *rgl2* and *sleepy1* but did not break the dormancy of *ga3ox1*. Exogenous GA treatment broke the seed dormancy of *ga3ox1* and *rgl2* but did not break the dormancy of *sleepy1* under normal temperature (Fig. 2). However, higher levels of *RGL2* mRNA accumulated in *sleepy1* (Fig. 5 and 7). These results suggest that RGL2 itself is not needed for the action of stratification.

RGL2, one of the DELLA proteins, has also been reported as a node of crosstalk between ABA and GA (Liu et al. 2016). The levels of both RGL2 and ABI5 proteins are positively regulated by ABA and negatively regulated by GA (Liu et al. 2016; Piskurewicz et al. 2008). XERICO, a RING-H2-type zinc-finger protein that affects ABA metabolism, is a direct target of DELLA repressors and is essential for GA signaling (Ariizumi et al. 2013). In this study, we found that seeds of the *sleepy1* mutant exhibited higher sensitivity to ABA, while those of the *rgl2* mutant exhibited lower sensitivity during imbibition (Fig. 5). The *cyp707a2* mutant accumulated higher endogenous ABA content and higher *RGL2* mRNA levels during imbibition (Fig. 7). Stratification breaks the dormancy induced by high levels of ABA from both exogenous and endogenous sources (Figs. 5, 6 and 7). Stratification does not override the inhibition of ABA on green cotyledon emergence (Figs. 1 and 6). These results indicate that ABA inhibits seed germination via the RGL2 pathway, while ABA-inhibited seedling development occurs through different pathways.

So, what is the target gene regulated by RGL2 and involved in ABA- and GA-regulated seed dormancy? It is well known that during cereal seed germination, some hydrolytic enzymes, including α-amylase, are secreted into the endosperm, where they hydrolyze starch and proteins to supply nutrients to the developing embryo (Weiss & Ori, 2007). The enzyme α-amylase is known to be activated by GA and suppressed by ABA, which can be used as a marker for starch hydrolysis (Liu et al. 2016). We also observed that GA and stratification increased starch hydrolysis by increasing the activity of α-amylase and the expression of the *AMY1*, *AMY2*, and *AMY3* genes, while ABA had the opposite effect (Fig. 3). Interestingly, these effects of ABA are overridden by stratification. The activity of α-amylase was also negatively correlated with the expression of *RGL2* in the *sleepy1* mutant and the *rgl2* mutant (Fig. 5). These results indicate that ABA regulates the activity of α-amylase and the expression of *AMY1*, *AMY2*, and *AMY3 *via an RGL2-regulated pathway under normal temperature conditions.

Under cold-pretreatment, the *cyp707a2*, *sleepy1*, and their double mutant *cyp707a2 sleepy1* exhibited comparably high ABA content and expression levels of *RGL2* and *ABI5* when compared to the control. Conversely, all three mutants exhibited increased starch hydrolysis by increasing the activity of α-amylase and the expression levels of *AMY1*, *AMY2*, and *AMY3* when compared to normal temperature (Fig. 6 and 7). These data suggest that stratification completely unlocks the inhibitions caused by ABA and RGL2 on starch hydrolysis.

As a physical stimulus, how does stratification modulate the RGL2/ABI5-mediated signaling pathway? Although this study did not directly elucidate its upstream sensing mechanism, we can propose several plausible hypotheses by integrating existing research. First, stratification may induce the expression of certain genes, such as GA biosynthetic genes, thereby increasing GA levels and subsequently participating in the recognition and degradation regulation of *RGL2*. Secondly, low temperature may trigger intracellular signal transduction by affecting cell membrane fluidity and cytoskeletal rearrangement, such as kinase cascade reactions that phosphorylate and modify key components in signaling pathways (Otani et al. 2024). Finally, epigenetic regulation may play a crucial role. Low temperature has been shown to induce genome-wide DNA methylation, histone modifications, and changes in non-coding RNAs. These alterations may directly modify the chromatin structure at the *RGL2* or *ABI5* gene loci or influence the expression of their upstream regulatory factors, thereby achieving transcriptional regulation of these two hub genes (Sacharowski et al. 2025).

**Fig. 8:**
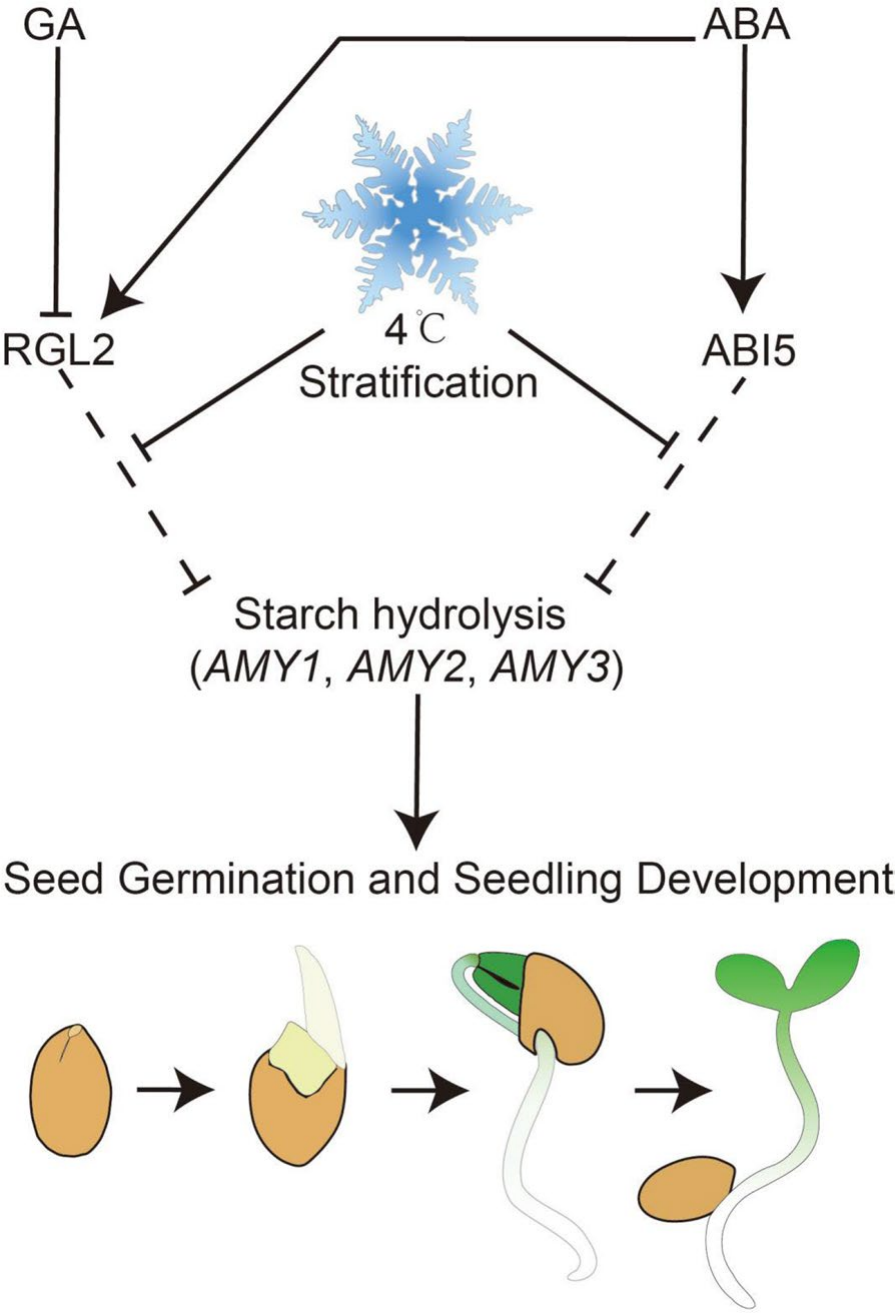
Model of seed germination control under stratification. Solid lines represent direct regulations or biochemical reactions. Dashed lines represent indirect regulations or proposed pathways

We propose a hypothetical model for the control of seed germination under cold-pretreatment, in which RGL2 acts as a node involved in both GA- and ABA-regulated seed dormancy (Fig. 8). Under normal temperature conditions, high levels of ABA signaling within the seed stabilize RGL2 and activate ABI5. RGL2 and ABI5 together form a critical inhibitory module that strongly suppresses the expression and activity of α-amylase genes, thereby preventing starch hydrolysis and maintaining seed dormancy. The GA signaling pathway is antagonized by RGL2-ABI5. Cold-pretreatment, on one hand, induces GA biosynthesis, and more crucially, effectively relieves the inhibition of α-amylase by RGL2-ABI5, thereby activating the starch hydrolysis process and driving seed germination and seedling development. This model elucidates the molecular mechanism by which cold-pretreatment promotes starch hydrolysis to break seed dormancy through the regulation of the hormone signaling network.

## Conclusion

In this study, we conclude that stratification effectively overcomes seed dormancy by orchestrating the RGL2/ABI5 inhibitory module from the expression of α-amylase genes. First, it triggers GA biosynthesis, which in turn relieves the repression of α-amylase expression mediated by the DELLA protein RGL2. Second, and more importantly, stratification overcomes the inhibitory effect of ABA on germination by uncoupling the RGL2/ABI5 regulatory module from its control over α-amylase activity. This allows for efficient starch hydrolysis and energy supply, even in the presence of high ABA levels. Our study reveals that stratification acts as a key environmental signal that reprograms the hormonal network, specifically by disabling the RGL2/ABI5 checkpoint, to promote seed germination.

## Materials and methods

### Plant materials

The Arabidopsis (*Arabidopsis thaliana*) (Col-0 and T-DNA inserted mutants from Col-0) plants were grown in a growth chamber with a 16—h photoperiod at a photoflux density of *c*. 200 μmol m^−2^ s^−1^ at a day temperature of 23℃and a night temperature of 20℃. To minimize the effect of seed maturation and storage conditions, plants of each genotype tested were grown in different sections of the same pot and seeds were harvested at the same time. Seeds were harvested in bulk 30 d after the petals appeared on the first flowers. These seeds maintained stronger dormancy. Only freshly harvested seeds were used to do the examination. The rest of seeds were stored at −80℃ Dormancy can be maintained for > 1 yr at −80℃.

### T-DNA insertion line

The seeds of *Arabidopsis thaliana cyp707a2*, *ga3ox1*, *sleepy1* and *rgl2* (SALK_083966, SALK_098513C, SALK_097562, SALK_024532C) generated by Salk Institute Genomic Analysis Laboratory (http://signal.salk.edu/) were obtained from the Arabidopsis Biological Resource Center (ABRC) (Ohio State University, Columbus, OH, USA). The seeds were planted on agar plates containing kanamycin and the kanamycin-resistant plants were transferred to soil. Seeds were harvested separately from individual plants. Subsequently, to confirm the mutant line as homozygous, PCR was performed with the genomic DNA of *cyp707a2*, *ga3ox1*, *sleepy1* and *rgl2* mutants using gene-specific oligonucleotides and LB primer (Supplemental Table 1).

### Generation of *CPY707A2* over-expressing line and *cyp707a2 sleepy1* double mutant

*CPY707A2* over-expressing line was obtained with the methods described by Liu et al. (Liu et al. 2009). *cyp707a2 sleepy1* double mutant was obtained by cross the mutant *cyp707a2* and *sleepy1*, then selected the double mutant at F_2_ generation.

### Germination assay

Fifty seeds were placed in 55—mm-diameter Petri dishes with three Whatman No.1 filter papers and 2.2 ml of sterile double-distilled water or treatment solutions. Plates were then placed in a 21 ℃ growth chamber without light for 24 h and transfer to continuous light at 100 μM m^−2^ s^−1^ at 21 ℃ for 7 days, if do the stratification treatment, plates were then placed in a 4 ℃ growth chamber without light for 24 h and transfer to continuous light at 100 μM m^−2^ s^−1^ at 21 ℃ for 7 days. The germination ratio was accounted after 7 days imbibition. The seeds were regarded as germination when radicle emerged. Experiments were performed in quadruple for each treatment.

### Extraction and determination of ABA

The method of ABA extraction and determination was modified according to previous research (Dobrev et al. 2005). Plant samples were ground to fine powder in the presence of liquid nitrogen. Then, 0.5 g powdered were extracted at 4 ℃ for 12 h with either 5 ml 80% methanol, and in both cases, before any extraction was performed, 2 ng [^3^H]-ABA (Perkin Elmer, BLU007H500UC) were added as internal standard to percentage recovery. After distilling methanol on a rotary evaporator, lipids were removed by partitioning the aqueous concentrate twice with 5 ml hexanes. The pH of the aqueous phase was adjusted to 2.5 with 6 n HCl and extracted three times with 5 ml ethyl acetate. The acidic fraction was dried with Concentrator plus at 30 ℃ (Eppendorf, German) and dissolved in 5 ml 50% methanol. Then the solution passed through a preconditioned Sep-Pak C18 column (Waters, American) and washed by 5 ml 50% methanol. The solution was dried with Concentrator plus at 30 ℃ and resolved with 55% methanol for analysis.

The method of LC/MS according to Hou et al. and modified (Hou et al. 2008). The mobile phases 0.05% (v/v) formic acid/water (solvent A) and 0.05% (v/v) formic acid/methanol (solvent B) were used in a gradient mode with the following conditions: time/concentration (min/%) for B: 0.0/55; 2/55; 10/90; 15/90; 22/55; 30/55. The chromatographic column we used is Agilent XDB-C18 (1.8 µm, 4.6 × 50 mm, USA).

The experiment was performed on a Finnigan LC–MS/MS system (Thermo Electron, San Jose, CA, USA) consisting of a surveyor autosampler, a surveyor MS pump and a Finnigan LTQ linear ion trap mass spectrometer equipped with an ESI source that was operated in negative mode. The SRM mode was used for the determination of the ABA, ABA were monitored at *m*/*z* transitions 263 → 153, 219.

### Quantitative analysis

Total RNA was isolated from seeds or leaves by RNeasy kit (Invitrogen). DNA impurities in the isolated RNA were digested before synthesizing the cDNA by adding DNase (Invitrogen) and incubated for 30 min at 37 ℃. DNase was then inactivated by incubating for 10 min at 65 ℃. Then 2 µg of RNA was reversed to cDNA with SuperScriptIII RTS First-Strand cDNA Synthesis Kit (Invitrogen). After that, the cDNA was diluted 10 times, and 4 μl cDNA was used to do the qRT-PCR. IQ™ SYBR Green Supermix (Bio-Rad) was used to do the qRT-PCR. *ACTIN 2* acted as the intramural standard. The qRT-PCR was executed with iCycle (Bio-Rad). The primes that were used in qRT-PCR are listed in supplemental Table 1.

### Measurement of α-amylase activity

The method of α-amylase activity measurement was modified according to previous research (Celińska et al. 2016). Extracts were prepared by grinding tissues in the cold extraction buffer composed of 50 mM Tris-HC1 (pH 8.0, 5 mM EDTA, 0.25 M SUC, and 10 mM DTT). The homogenate was centrifuged at 15,000 g for 10 min and the supernatant was used as the protein extract. Total amylase activity, measured as the increase in concentration of reducing sugars, was assayed with soluble starch as the substrate.

### Accession numbers

Sequence data from the article can be found in the GenBank data libraries or TIGR database (*Arabidopsis thaliana* Genome Project) under the following accession numbers: *RGL2*, AT3G03450; *ABI5*, AT2G36270; *AMY1*, AT4G25000; *AMY2*, AT1G76130; *AMY3*, AT1G69830; *CYP707A2*, AT2G29090; *GA3OX1*, AT1G15550; *SLEEPY1*, AT4G24210; *AAO3*, AT2G27150; *NCED6*, AT3G24220; *CYP707A1*, AT4G19230; *CYP707A3*, AT5G45340; *CYP707A4*, AT3G19270; *GA20OX1*, AT4G25420; *GA20OX3*, AT5G07200; *GA3OX2*, AT1G80340; *XTH5*, AT5G13870; *EXP2*, AT5G05290; *ACTIN2*, AT3G18780.

## Supplementary Information

Supplementary Material 1.

Supplementary Material 2.

## Data Availability

All data had been supplied in supplementary information.

## Abbreviations

ABA: Abscisic Acid
GA: Gibberellins
NO: Nitric oxide
ROS: Reactive oxygen species
PAC: Paclobutrazol
WT: Wild type
GRAS: GAI, RGA, and Scarecrow
RGA: REPRESSOR OF GA
GAI: GIBBERELLIC ACID INSENSITIVE
RGL1: RGA-LIKE1
RGL2: RGA-LIKE2
RGL3: RGA-LIKE3
ABI: ABA-Insensitive
