# Supplementary material for: Stratification overcomes ABA-mediated seed dormancy by uncoupling RGL2/ABI5 inhibition from α-amylase expression
Source: Stress Biol. 2026 Jun 22;6(1):43. doi: 10.1007/s44154-026-00312-6 (PMC13287508; doi:10.1007/s44154-026-00312-6)
Supplement: Supplementary file 1 — Supplementary Material 1. [file 44154_2026_312_MOESM1_ESM.docx]

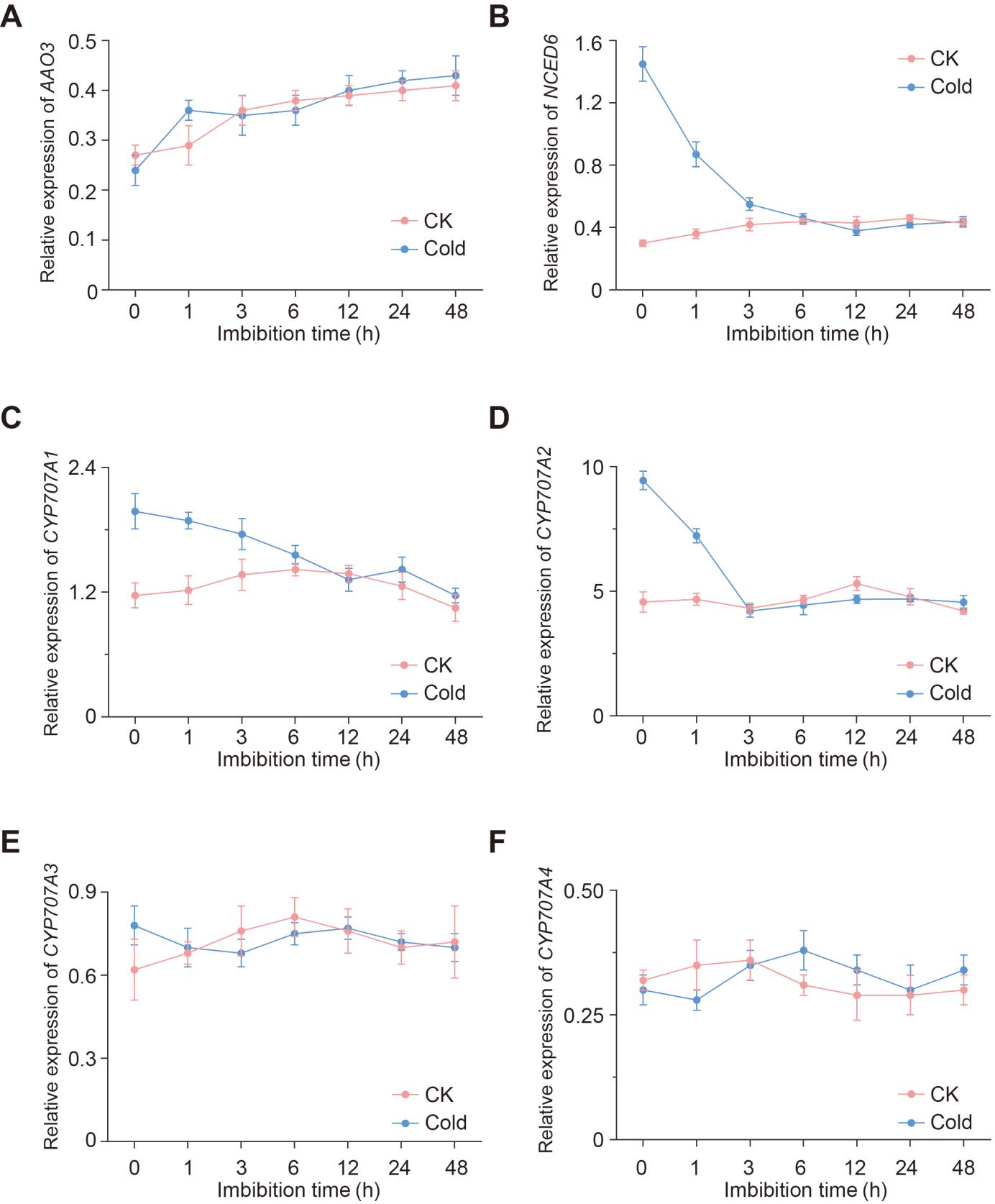


**Fig. S1** The expression of ABA related genes in seeds under stratification pre-treatment for different durations. Seeds were treated with (Cold) or without stratification (CK).


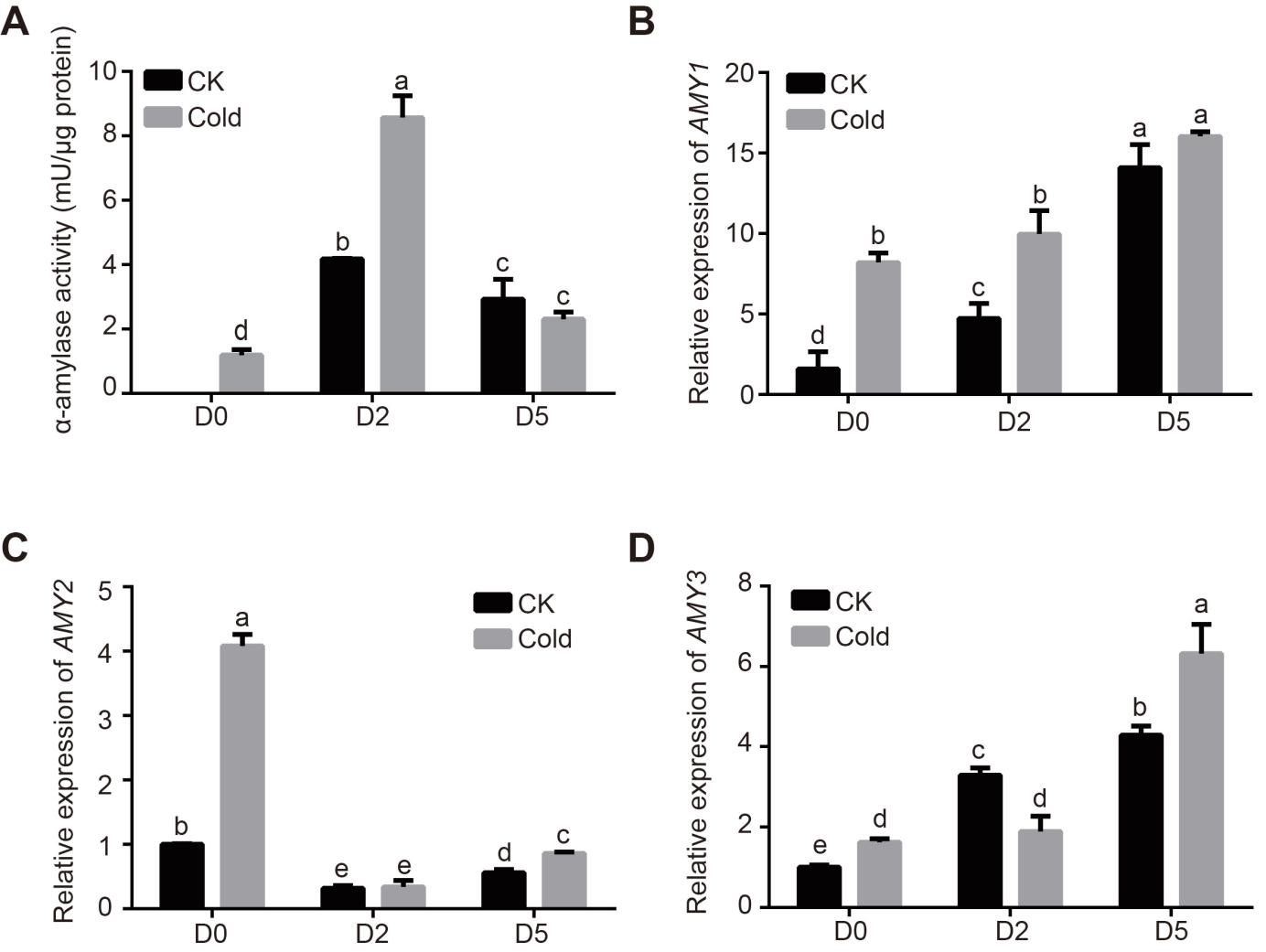


**Fig. S2** The effect of different development stages on α-amylase activity and their genes expression under 0.25 μM ABA. **A** The effect of D0, D2, D5 on the activity of α-amylase. **B** The effect of D0, D2, D5 on the expression of *AMY1*. **C** The effect of D0, D2, D5 on the expression of *AMY2*. **D** The effect of D0, D2, D5 on the expression of *AMY3*. Seeds were treated with (Cold) or without stratification (CK). The activity of α-amylase and the genes expression were analyzed after different development stages, D0 represents the 0th day of germination, D2 represents the 2th day of germination, D5 represents the 5th day of germination. Different letters above columns indicate significant differences (one-way ANOVA, *P* < 0.05).


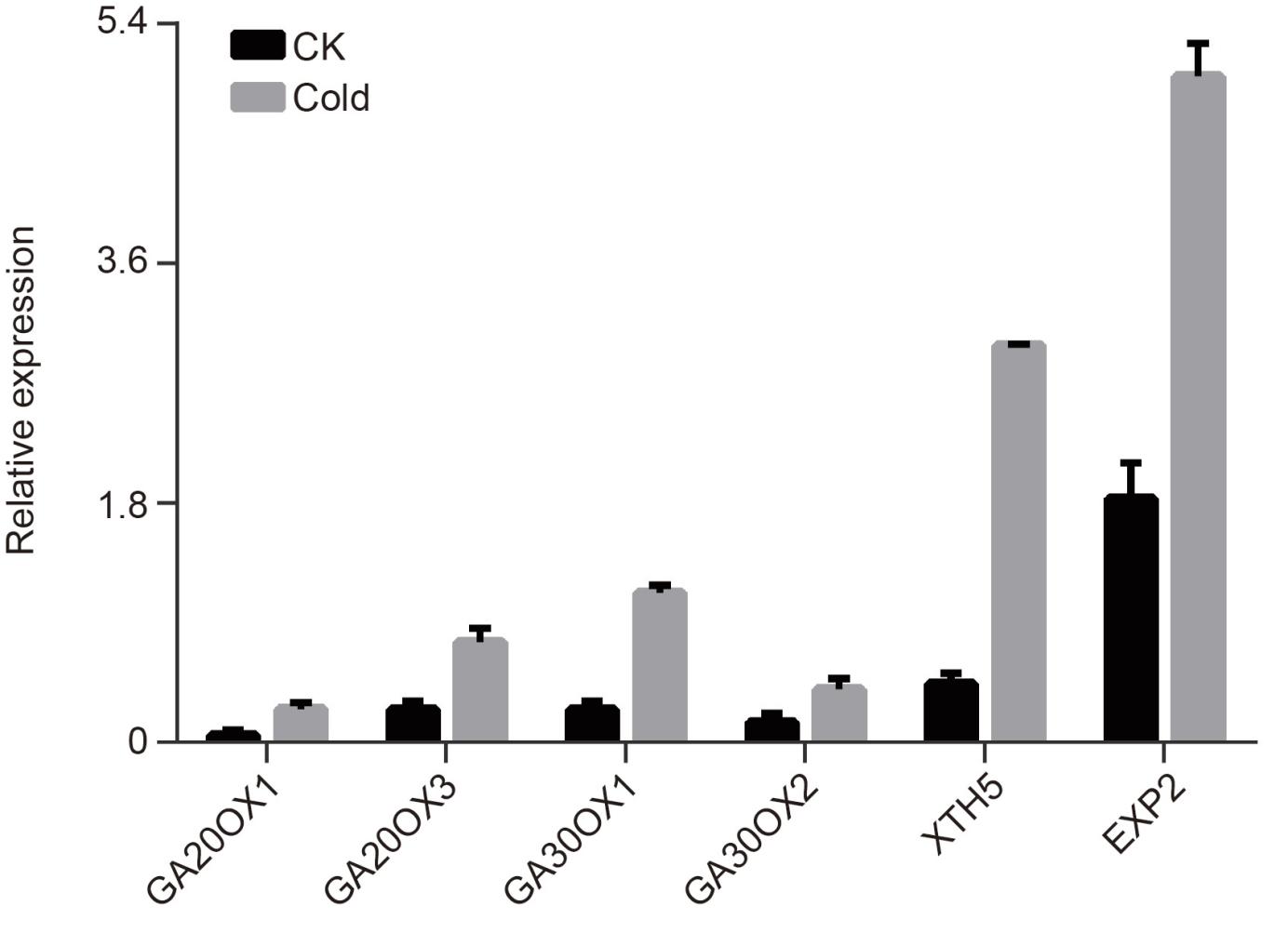


**Fig. S3** The expression of GA related genes in seeds under stratification pre-treatment. Seeds were treated with (Cold) or without stratification (CK). Different letters above columns indicate significant differences (one-way ANOVA, *P* < 0.05).


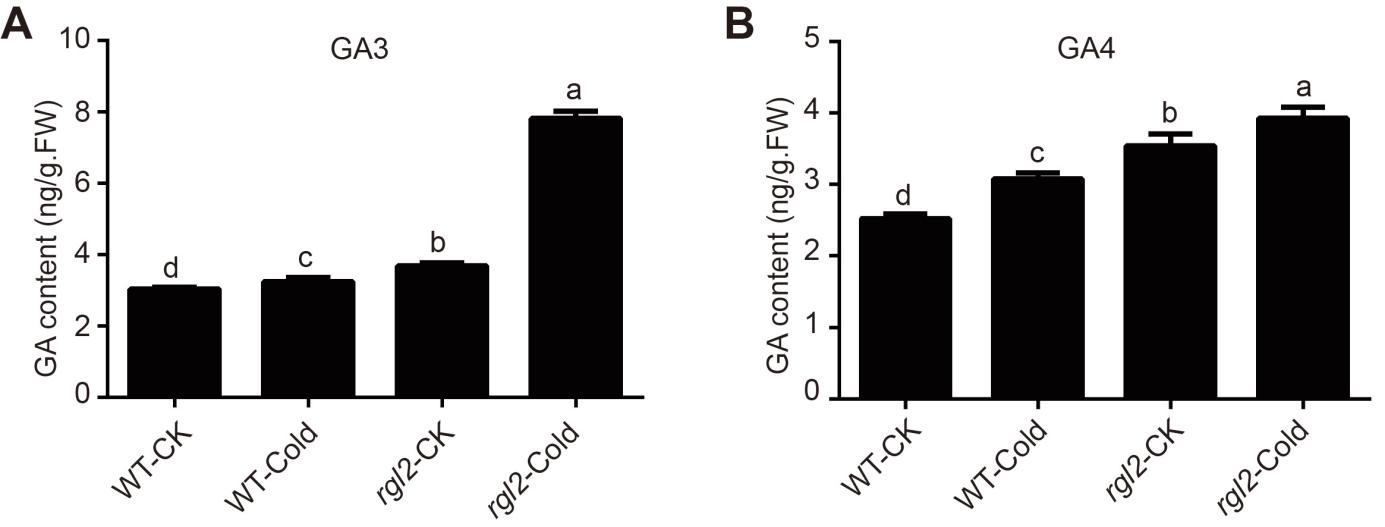


**Fig. S4** GA content under diverse stratification conditions. **A** GA3 content in different groups. **B** GA4 content in different groups. WT and *rgl2* seeds were treated with (Cold) or without stratification (CK). Different letters above columns indicate significant differences (one-way ANOVA, *P* < 0.05).


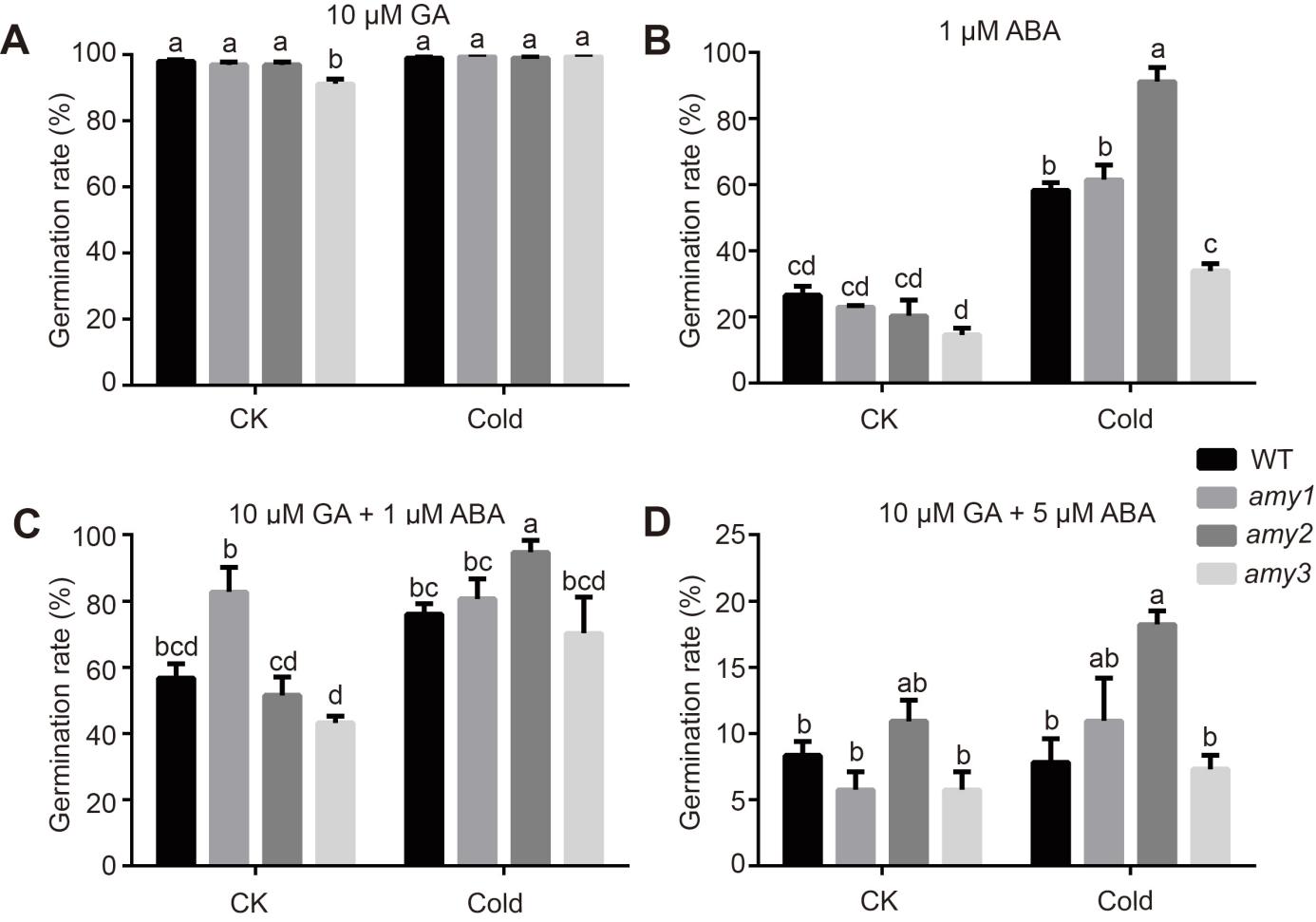


**Fig. S5** The effect of stratification on seed dormancy break in WT and *amy* seeds. **A** Germination rates of different genotypes after 6 days of 10 μM GA treatment. **B** Germination rates of different genotypes after 6 days of 1 μM ABA treatment. **C** Germination rates of different genotypes after 6 days of 10 μM GA and 1 μM ABA treatment. **D** Germination rates of different genotypes after 6 days of 10 μM GA and 5 μM ABA treatment. Seeds were treated with (Cold) or without stratification (CK). Different letters above columns indicate significant differences (one-way ANOVA, *P* < 0.05).


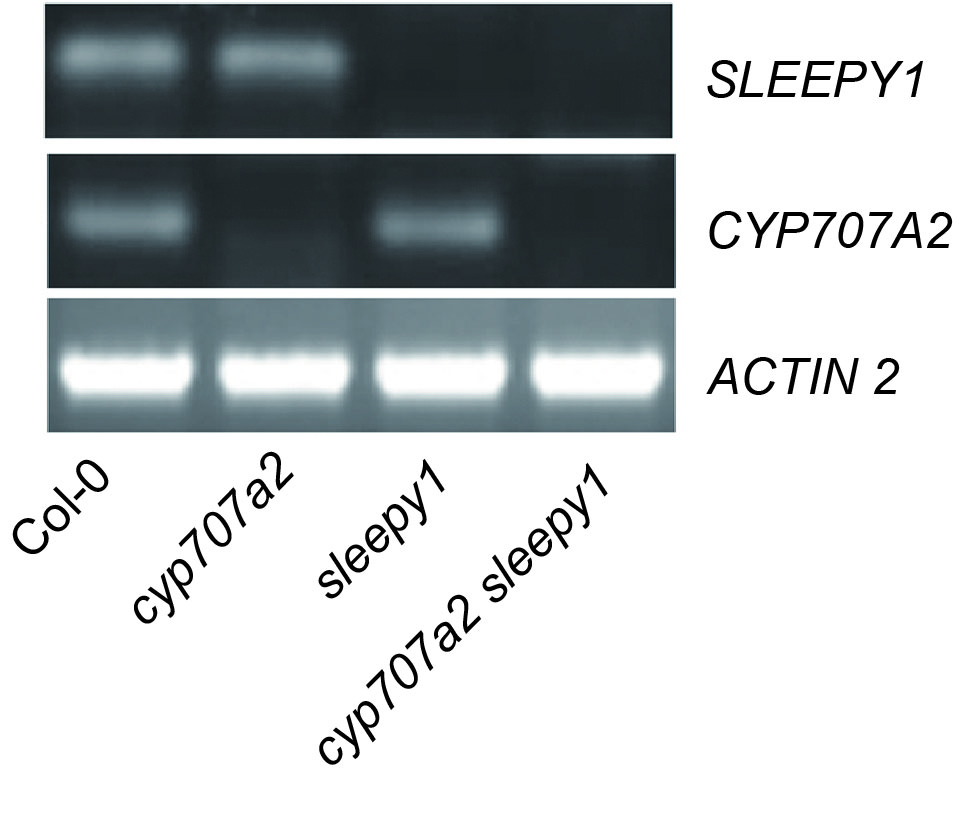


**Fig. S6** RT identification of different mutants.
